# Engineering neoantigen vaccines to improve cancer personalized immunotherapy

**DOI:** 10.7150/ijbs.76281

**Published:** 2022-09-01

**Authors:** Zaoqu Liu, Jinxiang Lv, Qin Dang, Long Liu, Siyuan Weng, Libo Wang, Zhaokai Zhou, Ying Kong, Huanyun Li, Yilin Han, Xinwei Han

**Affiliations:** 1 Department of Interventional Radiology, The First Affiliated Hospital of Zhengzhou University, Zhengzhou, Henan 450052, China.; 2 Interventional Institute of Zhengzhou University, Zhengzhou, Henan 450052, China.; 3 Interventional Treatment and Clinical Research Center of Henan Province, Zhengzhou, Henan 450052, China.; 4 Department of Gastroenterology, The First Affiliated Hospital of Zhengzhou University, Zhengzhou, Henan 450052, China.; 5 Department of Colorectal Surgery, The First Affiliated Hospital of Zhengzhou University, Zhengzhou, Henan 450052, China.; 6 Department of Hepatobiliary and Pancreatic Surgery, The First Affiliated Hospital of Zhengzhou University, Zhengzhou, Henan 450052, China.; 7 Department of Pediatric Urology, The First Affiliated Hospital of Zhengzhou University, Zhengzhou, Henan 40052, China.

**Keywords:** Neoantigen, Cancer, Vaccine, Immunotherapy, Personalized therapy

## Abstract

Immunotherapy treatments harnessing the immune system herald a new era of personalized medicine, offering considerable benefits for cancer patients. Over the past years, tumor neoantigens emerged as a rising star in immunotherapy. Neoantigens are tumor-specific antigens arising from somatic mutations, which are proceeded and presented by the major histocompatibility complex on the cell surface. With the advancement of sequencing technology and bioinformatics engineering, the recognition of neoantigens has accelerated and is expected to be incorporated into the clinical routine. Currently, tumor vaccines against neoantigens mainly encompass peptides, DNA, RNA, and dendritic cells, which are extremely specific to individual patients. Due to the high immunogenicity of neoantigens, tumor vaccines could activate and expand antigen-specific CD4+ and CD8+ T cells to intensify anti-tumor immunity. Herein, we introduce the origin and prediction of neoantigens and compare the advantages and disadvantages of multiple types of neoantigen vaccines. Besides, we review the immunizations and the current clinical research status in neoantigen vaccines, and outline strategies for enhancing the efficacy of neoantigen vaccines. Finally, we present the challenges facing the application of neoantigens.

## Introduction

Malignant tumors are the leading cause of mortality and remain a primary stumbling block to improving life expectancy worldwide 1. According to the 2022 Cancer Statistics, the number of new cancer cases and deaths from cancer in the United States is projected to be 1,918,030 and 609,360, respectively 2. Traditional cancer treatments, including surgery, radiotherapy, chemotherapy, and targeted therapy, have drawbacks. Surgical treatment is limited due to the possibilities of postoperative infections, tumor metastasis, and recurrence. Repopulation of cancer cells, resistance, and high recurrence rate are considered as main factors limiting the efficacy of radiotherapy 3-5. Chemotherapy outcomes are restricted by resistance, safe dosage, and unspecific cytotoxicity of chemotherapeutic drugs 6-8. Drug resistance demonstrates a dominant problem for the failure of targeted therapy 9. Besides, targeted therapy has certain requirements on the patient population, such as the compatibility of targets and the economic situation of patients 10. Furthermore, not all tumors have suitable targeted drugs. For example, developing targeted drugs for triple-negative breast cancer (TNBC) is problematic 11.

A major contribution to cancer immunotherapy has been made by immune checkpoint inhibitors (ICIs). ICIs activate the immune system and stimulate the production of antitumor immune cells once they release the inhibitory brakes of T cells 12. Presentation and recognition of neoantigens are crucial to the efficacy of ICIs 13. Neoantigens, known as tumor-specific antigens, differ from tumor-associated antigens. Neoantigens are only expressed in cancer cells but absent in normal cells, however, tumor-associated antigens exist both in cancer cells and normal cells. Thereby, neoantigens prevent “off-target” damage to normal tissues and are not subject to central or peripheral tolerance, making neoantigens attractive for personalized vaccines 14, 15. Neoantigen-specific T cells could be activated by sophisticated sequencing, recognization of individual mutations, computational forecasts of neoantigens, and vaccination of neoantigen vaccines 16. Cancer patients with the most abundant CD8+ T cell infiltration and the highest neoantigen number, but not either alone, have the longest survival. Meanwhile, long-term survivors exhibited sustained T cell reactivity to high-quality neoantigens. It is identified that neoantigen quality rather than quantity might guide the application of immunotherapies 17. In recent years, cancer vaccines have focused on neoantigens. Neoantigen vaccines furnish cancer patients with options and hopes, and show a bright future in different categories of cancers, including melanoma 18, non-small cell lung cancer (NSCLC) 19, glioma 20, and ovarian cancer 21.

In this review, we discussed the origin and identification of neoantigens, the immunological mechanism and the clinical application of neoantigen vaccines. Finally, we posed challenges to the utilization of neoantigens.

## The origin of neoantigen

### Single nucleotide variations

The somatic mutations that generated genetic variation within multicellular individuals were crucial to cancer occurrence and development 22. Parkhurst et al. 23 performed whole exon sequencing (WES) on tumors of patients with gastrointestinal cancer, defecting mutations ranging from 22 to 928 with a median of 114. They found that single nucleotide variations (SNVs) accounted for the majority of mutations and minority gene products encoded by somatic non-synonymous SNVs (nsSNVs) were immunogenic 23. Besides, 83% of patients possessed tumor-infiltrating lymphocytes (TILs) reactivity to neoantigens 23. Point mutation in the arginine 132 (R132) residue of *IDH1* was known as “hot spots” 24, serving as a potential immunotherapeutic target. IDH1 (R132H) was combined with the major histocompatibility complex (MHC) II allele human leukocyte antigen (HLA), whereby stimulated specific CD4+ T-helper-1 (Th1) cells to generate interferon-γ (IFN-γ). Further, a patient with IDH1 (R132H) glioma was detected to have IgG1 subclass-specific antibodies 25, anti-IDH vaccines might be a viable new treatment strategy. Additionally, the majority of diffuse gliomas showed a mutation at position 27 of histone H3.3 (H3.3K27M mutation) that changed lysine (K) to methionine (M) 26. Long peptide vaccines induced the expansion of specific T cell lines in progressive midline glioma with an H3.3K27M mutation. The H3.3K27M peptide bound HLA-A*02:01 with high affinity, subsequently connected with CD8+ T cells. Moreover, in HLA-A2+H3.3K27M+ glioma cells, T cell receptor (TCR)-transduced cells killed tumor cells in an antigen- and HLA-specific manner, which provided a powerful method for adoptive T cell therapy (ACT) of neoantigens 27. In addition, hepatocellular carcinoma (HCC) was a solid tumor with a low/moderate mutation load, and HCC neoantigens were rarely reported. Underlying neoantigens produced by nsSNVs in HCC were identified by WES and RNA sequencing. Most predictive peptides bound to HLA-A*02.01 and HLA-DRB1*01 representative class I and class II alleles and enjoyed immunogenicity, providing a novel strategy for HCC treatment 28. It was noteworthy that nsSNVs and neoantigen loads were associated with checkpoint inhibitor responses. ICIs were highly efficient in tumors with high nsSNVs load, such as melanoma, lung cancer, head and neck squamous cell carcinoma, and bladder cancer 29.

### Insertions and deletions

Insertions and deletions (Indels) gave rise to frame-shifting, formed a new open reading framework, and generated alien neoantigens. Across a pan-cancer cohort, renal cell carcinoma had the highest proportion and number of indels. Compared to nsSNVs, indels generated three times more predicted neoantigens as well as nine times more powerful mutant-binding neoantigens 30. Indels were ideal sources of tumor-derived neoantigens and capable of inducing multiple neoantigen responsive T cells while decreasing susceptibility to the tolerance due to the increased number of mutant peptides. Proverbially, overexpression of the epidermal growth factor receptor (EGFR) was connected with various cancers 31. The most frequent EGFR mutation was EGFR variant III (EGFRvIII), which arose from the deletion of 267 amino acids in exons 2-7 of the *EGFR* gene 32. Cancer patients expressing EGFRvIII produced specific humoral and cellular immune responses to EGFRvIII 33, suggesting that EGFRvIII was an immunogenic neoantigen. Furthermore, the microsatellite instability-high (MSI-H) tumor phenotype was attributable to defects in the DNA repair mechanism caused by a loss of mismatch repair activity. Variations in DNA length resulting from nucleotide repetition in microsatellite site units were a cardinal characteristic of MSI-H. Indels in MSI-H cancer formed some shared frameshift peptides that could combine with MHC I and activate CD8+ T cells, producing IFN-γ and tumor necrosis factor-α (TNF-α). Moreover, the frameshift mutations were “further from self” 34. The shared neoantigens resulting from frameshift mutations of MSI-H cancer patients extensively existed and owned strong immunogenicity, suitable for “off-the-shelf” design of cancer vaccines. Particularly, indel load was associated with increased cytotoxic T cell infiltration and superior ICI responses. The presence of indels was explicitly conjoined with significantly longer progression-free survival (PFS) and made a difference in overall response rates and disease control rates in NSCLC patients treated with ICIs, suggesting indels might play a predictive role in ICIs responses 35.

### Gene fusions

Gene fusions played a crucial part in tumorigenesis, which were related to chromosomal structural variations 36. In general, tumors with high somatic mutation burden exhibit a greater response to ICIs. However, it has been found that ICIs can also be effective in tumors with a low somatic mutation burden. Through further studies, researchers found the emergence of gene fusions led to the stimulation of cytotoxic T cell responses 37. Peptides that spanned two gene breakpoints were excellent sources of neoantigens. For instance, Yang et al.^36^ discovered the DEK-AFF2 gene fusion produced the mutant peptide DKESEEEVS, which bound to MHC I and stimulated T cell activation, generating IFN-γ and CD137 positive staining of CD8+ T cells. Additionally, peptides derived from MYB-NFIB or MYBL1-NFIB fusion gene could be treated as neoantigens in a group of adenoid cystic cancers of the head and neck. The fusion between *CBFB* and *MYH11* genes arising from inv (16) and t (16;16) was momentous in taking acute myeloid leukemia (AML) forward. CBFB-MYH11+ HLA-B*40:01+ AML cell lines and primary human samples were killed by clones of high-affinity CD8+ T cells segregated from healthy donors. High-affinity CBFB-MYH11 epitope-specific TCR transduced into CD8+ T cells performing anti-leukemia activity 38. These manifested that *CBFB-MYH11* fusion took the shape of a neoantigen, which had potential as a target for immunotherapy and provided evidence of principle for fusion-directed T cell immunotherapy of AML. Similarly, the amino acid sequence at the break/fusion point of the *EWS/FLI1* fusion gene was changed, forming a neoantigen. Functionally rejuvenated CTLs induced from pluripotent stem cells targeting the neoantigen encoded by *EWS/FLI1* fusion gene may be a promising approach for treating Ewing sarcoma 39. Nevertheless, the relationship between fusion protein and immunotherapy responses was indistinct, especially when the tumor mutational burden (TMB) of fusion-positive cancers was low 40.

### Others

DNA mutations were a perfect source of neoantigens, whereas changes in RNA splicing led to the generation of new epitopes in the same way. Alternative splicing was a regulatory mechanism whereby a single gene produced multiple mRNA transcripts, significantly expanding proteome diversity 41. Disruption of splicing mechanisms affected transcription and was capable of resulting in disease. Pharmacological modulation of RNA splicing could produce true neoantigens that inhibited tumor growth and augmented checkpoint blocking in a manner reliant on peptides present on host T cells and tumor MHC I 42. Intron retention (IR) was the failure of the spliceosome to remove specific introns from the former mRNA molecule, allowing them to remain in the mature polyadenylated mRNA. IR was a significant source of multiple myeloma (MM) neoantigens, and newly diagnosed MM samples showed more IR events in comparison with normal plasma cells 43. Patients with MM had poor overall survival when they had high IR neoantigen loads. Besides, endogenous retroelements, HLA-somatic mutation-derived antigens, post-translational TSAs, and viral-derived cancer antigens (for example, HPV and EBV) are classes of surrogate neoantigens 44. For instance, dysregulation of protein citrullination was connected with autoimmune diseases. Citrullinated peptides could bind to MHC II, promoting the autoimmunity of rheumatoid arthritis and inducing a B cell response 45, 46. Recently, citrullinated peptides targeting vimentin and α-enolase have been certified to have anti-tumor immune effects by triggering a CD4+ T cell response, building evidence for the potential immunogenicity of protein citrullinated in tumors 47, 48. Likewise, Hiroyuki et al. 49 demonstrated that the protein citrulline was a source of cancer neoantigens.

## Identification and prediction of neoantigens

Although neoantigens were weakly expressed, recent advances in mass spectrometry (MS), exosome, and bioinformatics offered powerful tools for mining sparse samples 50. Somatic mutations were recognized using WES of tumor and normal cell DNA, followed by prioritization of mutated alleles using RNA sequencing, and the binding affinity between the putative neoantigen and MHC was predicted on an electronic computer subsequently^18^, ^51^. Though RNA sequencing had high false-positive and false-negative rates, WES lacked coverage and variant allele reads in the tumor. Nevertheless, combining the two was an effective and economical approach to predicting neoantigens, detecting about 70% of neoepitope candidates with high expression and rich mutant transcripts 52. Next, the IC50 value or percentile rank score was extensively used for affinity prediction. IC50 values reflected predictions of direct binding affinity, and peptide thresholds <500 nM were applied to identify compounds most likely to bind HLA. While the percentile rank represented relative neoantigen binding affinity, a rank score ≤2 was accustomed to selecting potential neoantigen binders 53. Unlike HLA I, the peptide-binding groove of HLA II was open to accommodate longer peptides. The flanking amino acids of the core binding sequence could affect the binding affinity 54. In addition, HLA II peptide processing was extraordinarily intricate and obscure, posing a challenge to the development of algorithms 55. Moreover, the prediction of neoantigens included other biological features other than gene expression and MHC affinities, such as mutation clonality, dissimilarity to self, and TCR recognition 56. A powerful way to discover neoantigens was a combined genomic, proteomic, and immunopeptidomics approach 57. Currently, various types of software applications are capable of discerning neoantigens. The positive predictive value of HLA antigens was increased up to ninefold through the EDGE model which used genomic dataset and HLA peptide MS data rather than HLA-peptide binding affinity data 58. Moreover, the features based on immunopeptidomics MS increased neoantigen prioritization up to 50% 59. Notably, MS provided posttranslational modification information, for example, glycosylation and phosphorylation 60, 61. Structural parameters could improve the prediction of immunogenic neoantigens 62. The convolutional neural network (CNN) was a particular type of deep neural network, more opportune for studying peptides whose amino acid spatial positions were critical for binding 63. A deep CNN named Antigen Presentation Prediction Model outperformed the means proposed by the immune epitope database on specificity and positive predictive value 64. Beyond that, the binding affinity to the same register was reduced by nearly 70 times in consideration of the proximal variation. Thereby, some weakly binding candidate peptides could be unsuitable 65. It was essential to achieve accuracy in predicting neoantigens during the development of neoantigen vaccines. However, neoantigen prediction tools are currently limited to SNVs, and few tools are available to predict mutations other than SNVs in neoantigens 66. Therefore, the development of bioinformatics is indispensable to improving the identification and evaluation of neoantigens.

## Neoantigen vaccines

**Figure 1:**
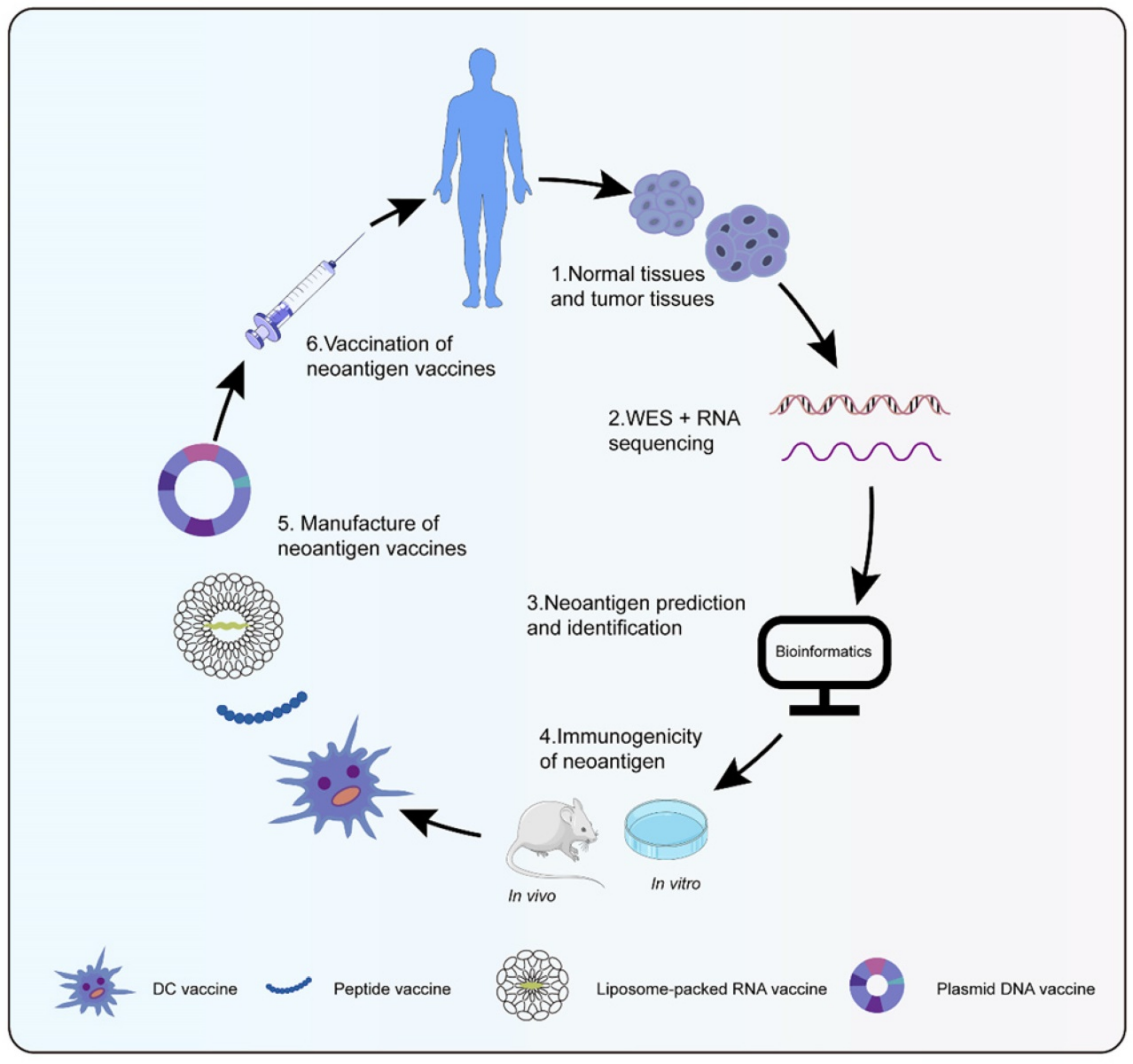
** Preparation of neoantigen vaccines.** Obtain normal tissue and tumor tissue from cancer patients, then perform whole exons and RNA sequencing to detect mutations. Bioinformatics technology screen out candidate neoantigens. Immunogenic neoantigens are identified by *in vivo* and *in vitro* experiments. At last, various types of neoantigen vaccines are prepared to treat cancer patients.

Neoantigen vaccines were highly personalized, whose process from tumor specimen collection, sequencing, and bioinformatics analysis to vaccine preparation generally took 3 to 5 months (Figure 1) 67. Cancer vaccines were designed in four broad types: peptides, DNA, RNA, and dendritic cell (DC) (Table 1). Peptide vaccines, the most pervasive form of the vaccine, consisted of recombinant or purified proteins. Peptide vaccines composed of 20-30 amino acids might be processed and presented by antigen presentation cells (APCs) as a matter of priority 68. After being cleaved by the immunoproteasome and antigen processing, short peptides (typically 9-11 amino acids in length) were affixed to MHC I, while long peptides (typically 14-16 amino acids in length) were attached to MHC II 69. Id22, a peptide vaccine, was efficient enough to protect mice from the growth of MM, which induced antigen-specific CD4+ Th activity and anti-tumor immunity 70. Meanwhile, an IDH1(R132H)-specific peptide vaccine (IDH1-vac) was equipped to engender specific Th cell responses arresting the growth of IDH1 (R132H) + tumors in mice 71. Nonetheless, peptide vaccines were restricted as a result of their inimitable peptide epitopes, low molecular weight, simple degradation, and short half-life 72, 73. Plasmid DNA vaccines represented another attractive approach for personalized vaccination because of fabrication easiness and low cost 74. The probability of integration between host genome and DNA vaccines was extremely low, even lower than spontaneous mutations 75. Robust antigen expression, processing, and presentation were in demand for the success of DNA vaccines. Tondini et al. 76 evaluated an optimized polyepitope DNA vaccine in murine melanoma. Vaccination with DNA vaccine was capable of eliciting T cell responses. Moreover, the combination of DNA vaccination and anti-PD-1 treatment suppressed tumor growth. RNA-based vaccines became a hotspot in the context of the coronavirus disease-2019 pandemic. Unlike plasmid DNA, RNA avoided possible insertion mutations and aberrant transcription. Additionally, the risk of side effects was reduced for fewer constituents to transform RNA into protein and biodegradation of RNA 77. Compared with peptide vaccines, RNA also had distinct advantages such as low synthesis expenses, short synthesis cycle, simultaneous encoding of multiple antigen sequences, and lack of MHC haplotype restriction. Effective delivery of RNA into cells was essential to RNA vaccines. DP7-C could convey antigens into cells to its advantage by caveolin- and clathrin-dependent pathways 78, 79. As a carrier of mRNA, DP7-C-modified DOTAP liposomes were more efficient in transferring mRNA into different types of DCs, which stimulated the maturation of DCs, production of CD103+ DCs (contributing to antigen presentation), and secretion of proinflammatory cytokine 80. The principle behind making DC vaccines was simple. DCs were transferred back into the patient after isolation and cultivation of patient DC progenitors and loading of tumor antigens. Henceforth, specific T cells stimulated by DCs could engage in anti-tumor responses. Due to their low toxicity, lack of invasive procedures, and potential long-term effects, DC vaccines were a particularly attractive immunotherapy option 81. However, the efficacy of DC vaccines was confined to advanced recurrent patients 82. Sipuleucel-T was the first DC vaccine for prostate cancer approved by the United States Food and Drug Administration 83. Different immune modes may produce distinct therapeutic effects and immune responses despite the same antigen. Neoantigen-pulsed DC vaccines were superior in immune and anti-tumor effects in contrast with the neoantigen-adjuvant vaccines 84. In summary, a new era of neoantigen vaccines was coming; additional experiments were in need for further application of neoantigen vaccines.

## Neoantigen vaccines and immune

### Immune responses

**Figure 2:**
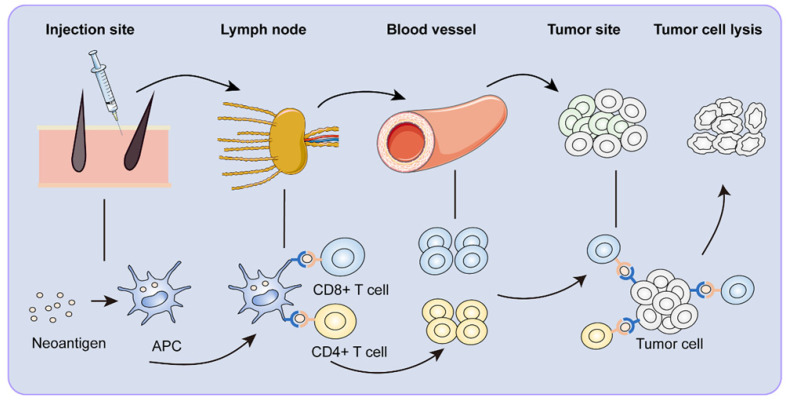
** Immune response to neoantigen vaccines.** After vaccination, neoantigen vaccines are first recognized by APCs around the injection site. The APCs reach the draining lymph nodes through lymphatic vessels. In the lymph node, neoantigen-specific T cells are activated. Activated CD8+ and CD4+ T cells clone and arrive at the tumor site with the circulatory system, specifically killing tumor cells.

ICIs have revolutionized cancer therapy. However, microsatellite stable colorectal cancer was not sensitive to ICIs because of its low mutation rate and stable microsatellites. In contrast, neoantigens appeared to offer promising immunotherapeutic potential 85. Therapeutic cancer vaccination was designed to produce a robust and sustained anti-tumor immune response 86, 87. For the perceived advantages of neoantigen, neoantigen vaccines have developed expeditiously in recent years. Neoantigen vaccines underwent a series of complex maturation processes to become functional (Figure 2).

As generally believed, CD8+ T cells were a central participant in killing tumor cells. CD8+ T cells either took place directly via synaptic exocytosis of cytotoxic granules that contained perforin and granzymes into the target cells or caused the indirect destruction of cancer cells through secreting cytokines, such as IFN-γ and TNF. CD40, CD70, and CD80/86 were of great importance in the activation and persistence of T cells mediated by DCs. Chemokine receptor CX3CR1 was a marker of effector T cell differentiation 88, 89. However, a recent study found the anti-tumor efficacy of neoantigen/toll-like receptor 3 (TLR3)/CD40 agonist vaccine and neoantigen-specific CX3CR1+ CD8+ T cell generation relied on CD40 and CD80/86, rather than the CD70 signaling pathway 90. To induce cytotoxicity and neoepitope-specific CD8+ T cell responses, therapeutic vaccines required a conjoined helper epitope, such as P30 91. Besides, B cells and CD4+ T follicular helper cells synergically promoted the anti-tumor CD8+ T cell responses 92. Nevertheless, without CD8+ T cells targeting the same antigen, CD4+ T cells have been proven to protect against tumor growth. The new epitope-specific TCR transgenic mice rejected MM cells. In the absence of CD8+ T and B cells, transgenic mice still had protection 93, demonstrating that neoepitope-specific CD4+ T cells offered sufficient production. These studies were the first to show that neoantigen-specific CD4+ T cells could repel tumor cells. Subsequent studies found that the transgenic mice also rejected B lymphoma cells 94. Tumor cells were killed indirectly by CD4+ T cells via cytotoxic macrophages depending on the inducible nitric oxide synthetase way 95. Furthermore, neoantigen vaccine-induced CD4+ T cells impacted the function of CD8+ T cells. On the one hand, they activated CD8+ T cells responses against non-vaccine, tumor-associated antigens. On the other hand, they promoted effector memory CD8+ T cells migrate to the tumor microenvironment (TME) 70, 96. CD4+ Th cells could facilitate activation of CD8+ T cells by licensing DCs through CD40/CD40L interactions 97. DCs' interaction with CD4+ T cells stimulated them to produce cytokines like IL-15 and increased CD80/86 and CD70 expression, which provoked a potent response from CD8+ T cells 98. Besides, CD4+ T cells could eliminate cancer via a cytotoxic phenotype mediated by perforin and granzyme B 99.

In addition, the analysis of immunogenicity and immunological characteristics in clinical trials provided a theoretical basis for the specific T cell responses of neoantigen vaccines. Keskin et al. 100 administered personalized neoantigen vaccination to 10 patients with glioblastoma. CD4+ and CD8+ T cells expressed cytotoxic markers (PRF1, GZMA, and GZMK), and a few CD4+ T cells were Tregs, co-expressing IL2RA and FOXP3. Most CD4+ and CD8+ T cells expressed at least one effector cytokine (IFNG, IL2, or TNF) 100. Co-inhibitory molecules (TIM-3, TIGIT, PD1, CTLA4, and LAG3) were also discriminatively expressed in CD4+ and CD8+ T cells. Moreover, peripheral blood neoantigen-specific T cells could migrate to intracranial glioblastoma 100. In another clinical trial, eight patients with surgically resected stage IIIB/C or IVM1A/B melanoma were treated with neoantigen vaccine. Transcriptional status analysis at diverse vaccination stages demonstrated that the differentiation state associated with T cells underwent a marked shift from infantile to effector, apoptosis, and memory 101. TCR sequence became abundant after inoculation. Furthermore, neoantigen vaccination expanded pre-existing T cell populations specific to neoantigens and resulted in a broader repertoire of novel T cell specificities, tipping the intra-tumoral balance in favor of better tumor control 18. Taken together, these studies provided valuable insights into the immunogenicity of neoantigen vaccines and their therapeutic potential.

### Immune escape

**Figure 3:**
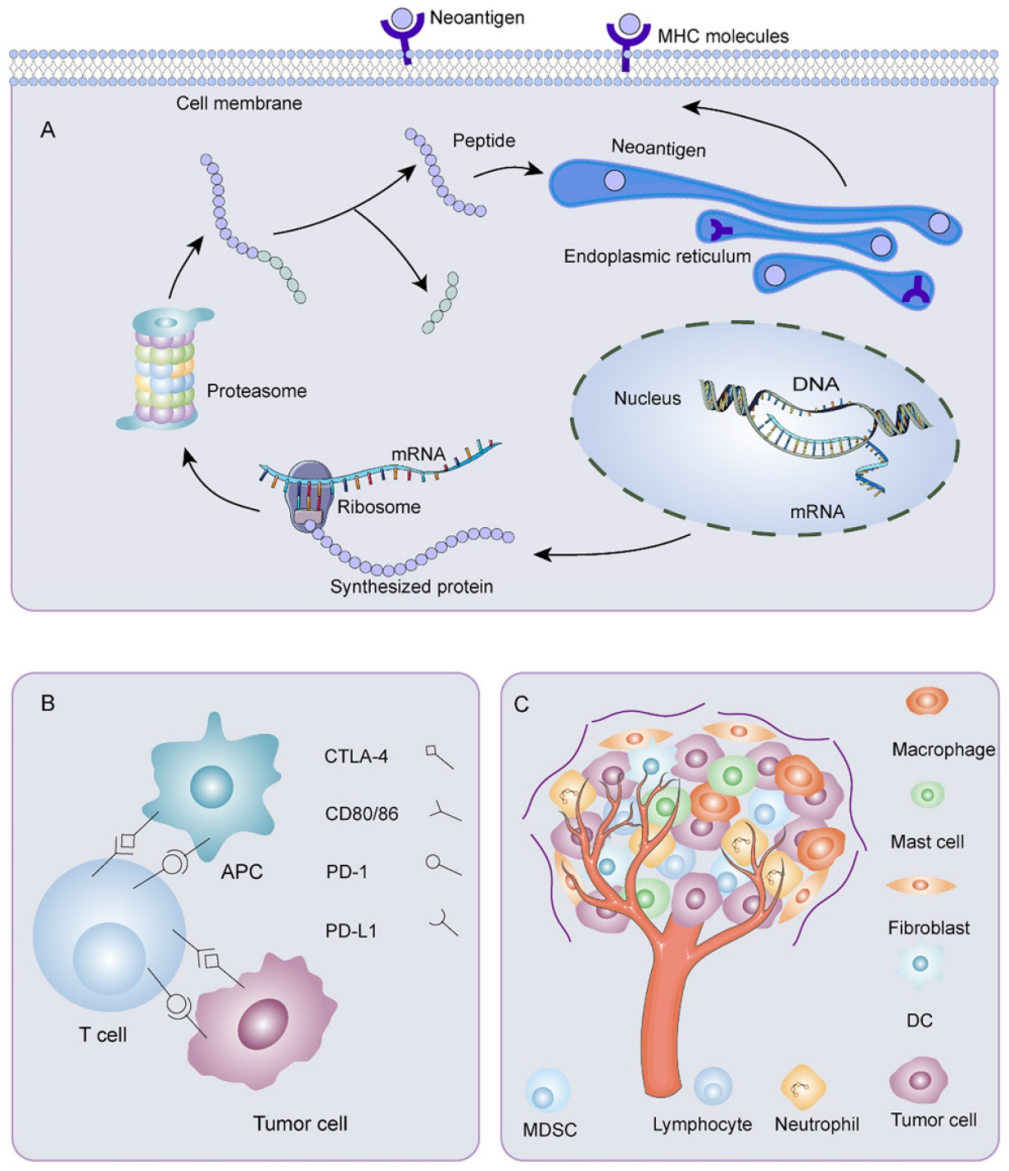
** Mechanisms of immune escape. A)** Mutant gene sequences are transcribed and translated into mutated proteins. After processing, the mutant peptides enter the endoplasmic reticulum. They bind MHC I or MHC II to form peptide-MHC molecular complexes, which are transported to the surface of tumor cells. Any abnormality in the process of neoantigen processing and presentation could result in immune escape. **B)** Overexpression of immune checkpoints restrains anti-tumor immune responses. **C)** Various components of tumor microenvironment are associated with the occurrence of immune escape.

Immune escape was a crucial problem affecting the effectiveness of neoantigen vaccines (Figure 3). The reduced neoantigen expression was an immune escape mechanism 102. DNA copy number variation induced by chromosomal instability may incite loss of neoantigens. Promoter hypermethylation caused preferential inhibition of genes containing neoantigen mutations, a latent mechanism for neoantigen deletion in the transcriptome 103, 104. Post-translational mechanisms and the silencing of genomic segments encoding neoantigens at the epigenetic level may also be instrumental in deleting neoantigens 103. Before being recognized by specific immune cells, neoantigens passed through a complicated intra-cellular mechanism involving the proteasome. Non-synonymous somatic mutations altered the chemical composition of amino acid sequences. As a result, the proteasomal cleavage properties varied on account of different cleavage preferences for basic, acidic, or hydrophobic amino acids. These may bring on ineffective neoantigen-specific T cell activation owing to a loss of binding affinity between the neoantigen and the MHC I complex or alteration of the complex's affinity for binding to the TCR. Lung cancer 105 and advanced bladder cancer 106 had a latent immune escape mechanism by altering proteasome antigen processsing. Meanwhile, HLA loss or mutation affected the stability of MHC and production of HLA enhancers, thus, disrupting antigen presentation, which provided an alternative mechanism for immune evasion 103, 107, 108. HLA I deficiency was more frequent in advanced tumors than early tumors, and the degree of infiltration of CD8+ T cells was noticeably lower in HLA I deficient tumor areas than in HLA I preserved tumor areas 109. Proteins were crucial for the maturation and exposure of antigens, such as antigen peptide transporter-2 (TAP2), beta2-microglobulin, and chaperone molecules, including calreticulin, ERp57 calnexin, and tapasin 110. Alterations in TAP1/2 interfered with antigen-presenting. The deletion of the beta2-microglobulin gene hindered immune surveillance of tumors 111. For example, in tumors with extrachromosomal DNA (ecDNA), immune cell infiltration and cytotoxic T cell activity were reduced. Through gene set enrichment analysis, expression of MHC-related genes decreased, which may be a dormant immune evasion mechanism of cancer with ecDNA 112. Moreover, impaired DC cross-priming resulted in site-dependent immune escape. CD40 agonist enhanced cross-presentation and restored immune control by promoting T cell initiation and expanding response through epitope diffusion 113. Besides, the exhaustion of T cells posed a significant challenge to antitumor immunity 114. Depleted T cells exhibited a progressive loss of effector functions, multiple inhibitory receptor expression, dysregulated metabolism, unsatisfactory memory recall response, and dysfunctional homeostasis, contributing to ineffective cancer control 115. Furthermore, immune checkpoints such as CTLA-4, PD-1, PD-L1, and PD-L2 were related to physiological self-tolerance. However, overexpression of immune checkpoints restrained anti-tumor immune responses. Meanwhile, TME, consisting of tumor cells, immune cells, interstitial tissue, and extracellular matrix, was inextricably linked with the development, invasion, and metastasis of tumors. The presence of tumor-associated fibroblasts 116, tumor-associated macrophages 117, tumor-associated mast cells 118, Tregs 119, and bone marrow-derived suppressor cells (MDSC) 120 helped tumor cells escape immune killing. In recent years, it has been found that tumor-associated neutrophils also had several connections to immune escape. In ovarian cancer, tumor-associated neutrophils coordinated intra-tumoral IL-8-driven immune escape through activation of Jagged2 121. In breast cancer, tumor-associated neutrophils advanced T cell immunosuppression through PD-L1 122. Metabolic enzymes like indoleamine 2, 3-dioxygenase 1 123, serine/threonine-protein kinase 1 124, and arginase 1 125 created a tolerant microenvironment. In addition, immunosuppressive cytokines such as IL-6 126, IL-4 127, and transforming growth factor-β (TGF-β) 128 acted analogously.

## Neoantigen vaccine and clinical application

### Monotherapy

Given the promising results from preclinical studies, numerous clinical trials of neoantigen vaccines are in progress (Table 2). The high recurrence rate after radical resection was the primary reason for the unfavorable prognosis of HCC patients. Transcatheter arterial chemoembolization (TACE) was an effective strategy for preventing recurrence. Cai et al. 129 subcutaneously injected neoantigen peptide vaccine into ten patients undergoing radical surgical resection and prophylactic TACE therapy. The median recurrence-free survival of the five patients with responsive neoantigens was considerably longer than those with non-responsive neoantigens or with only the initial vaccine. Notably, two patients maintained a relatively powerful neoantigen-specific immune response for ten months. In a clinical trial, 22 patients with advanced malignant tumors were treated with iNeo-Vac-P01, containing 5 to 20 peptides with 15 to 35 amino acids. Twenty participants experienced no or mild adverse reactions, while two had severe acute allergic reactions, showing a 71.4% disease control rate and a median PFS of 4.6 months. This trial also acknowledged that the mutations of genes and variations of copy number were predictive of the immune responses 130.

Nucleic acid-based vaccines could convey DNA or RNA encoding targeted epitopes to prevent and treat infectious diseases and cancer. Rather than targeting cancer neoantigens, most DNA vaccines focused on tumor-associated antigens, such as mammaglobin-A in breast cancer 131, HPV E6, and E7 in cervical cancer 132, and HER2/CEA in solid cancer 133. Clinical trials of neoantigen DNA vaccines in glioblastoma, TNBC, and pancreatic cancer are ongoing. In TNBC patients, neoantigen DNA vaccination was conducive to a robust immune response and prolonged PFS 134. Cafri et al. 135 recognized specific immunogenic mutations expressed in tumors, constructing a neoantigen vaccine called mRNA-4650, including up to 15 HLA I candidate neoantigens. Four patients with metastatic gastrointestinal cancer received the vaccine. Although no clinical response was observed, vaccine-induced CD4+ and CD8+ T cells were detected in three patients. RO7198457, an RNA-lipoplex vaccine, encoded up to twenty neoantigens. In phase Ib trial, it was single-tested in 29 patients with advanced solid tumors, including NSCLC, colorectal cancer, melanoma, and breast cancer. Achieve an objective response rate of 4% and a disease stabilization rate of 40%, slightly lower than the combination of RO7198457 and anti-PD-L1 antibody atezolizumab 136.

DCs were the dominant focus of cancer vaccines as antigen delivery carriers. In addition to acting as vaccines alone, various DNA, RNA, and peptides could be loaded onto DCs. The first neoantigen DC vaccine embarked on testing in 2015 137. In this trial, genomic analysis and computer simulation of neoantigen prediction were performed on three patients' surgically removed tumor tissues. Seven neoantigens appending to HLA-A*02:01 were screened out and incorporated into DC vaccine formulations together with melanoma gp100-derived peptides. T cell-triggered immune responses were enhanced, and all three patients survived without autoimmune adverse reactions. Furthermore, DC vaccines facilitated the expansion of the TCR repertoire of highly diverse neoantigens. Ding et al. 138 tested personalized neoantigen pulsed DC vaccine in 12 advanced lung cancer patients. After treatment, all adverse events were grade 1-2, and no delays due to toxic reactions to drugs occurred. Besides, they reached a 25% objective effectiveness rate, a 75% control rate for disease, a 5.5-month PFS, and a 7.9-month overall survival. Remarkably, a patient with extensively metastatic lung adenocarcinoma who had failed three treatments, including a PD-1 inhibitor, was treated with the vaccine with almost no metastatic lymph nodes, partial metastatic lesions, and a reduction in tumor target lesions.

In general, initial results suggested that peptide-, nucleic acid-, DC-based neoantigen vaccines were safe and immunogenic, revealing encouraging prospects as a feasible approach to cancer treatments and bringing prospective benefits to patients.

### Combine with other therapies

Neoantigen vaccines could stimulate an autoimmune response. However, the effectiveness of neoantigen vaccines was confined, which was attributable to the lack of appropriate immune stimulation and the impact of the immunosuppressive TME. ICIs boosted T cell responses and conduced to prolonged survival in patients with previously untreatable cancers 139-141. Although ICIs have been successful in numerous advanced malignancies, they were only effective in a small number of treated patients 142-144. Preclinical studies have shown that combining neoantigen vaccines and ICIs exhibited different T cell phenotypic characteristics, enhanced immune responses, and appreciably increased the total number of TCR clones 145-147. The combination therapy also upregulated the expression of genes linked to T cell activation and effector function. Neoantigen vaccines were expected to work synergistically with ICIs to reverse tumor-induced immunosuppression, contributing to clinical benefits. The first combination of an individualized neoantigen vaccine (NEO-PV-01) with PD-1 inhibition was reported in 60 patients with high TMB metastatic tumors, including melanoma, NSCLC, and urothelial carcinoma of the bladder. The objective response rates were 59%, 39%, and 27%, with median PFS of 23.5 months, 8.5 months, and 5.8 months. Meanwhile, the response to combination therapy was influenced by TMB and epitope quality 148. Ott et al. 18 administered neoantigen vaccines to six melanoma patients, four free of recurrence at 25 months. Two sufferers received anti-PD-1 therapy after recurrence and had complete tumor regression. Some researchers treated twenty patients with unresectable advanced tumors with neoantigen vaccine in combination with pembrolizumab. Six clinical responses have been reported, including two of the 12 individuals who previously received ICIs 149. These studies provided forceful support for the combined use of neoantigen vaccines and ICIs.

ACT targeted and killed tumor cells with the patients' T cells, which could promote the active invasion of T cells into tumor tissues and delay tumor progression. Therapeutic neoantigen vaccines and ACT transfer have shown propitious initial results. Tanaka et al. 150 sequenced the whole-exome and transcriptome of patients with the myelodysplastic syndrome to identify neoantigen candidates, then prepared T cells for specific neoantigen and injected them into the patients. No dose-limited toxicity or cytokine release syndrome was observed, and no apparent autoimmune response occurred. Kristensen et al. 151 performed ACT in patients with melanoma, finding that the number and frequency of new epitope-specific T cells in TILs infusion products were associated with improved clinical outcomes.

Furthermore, some preclinical studies have shown that neoantigen vaccines worked better when combined with other therapies. Tumor clearance after radiotherapy and chemotherapy was the induction of immunogenic cell death and the release of damage-associated molecular patterns 152. Chemotherapy or radiotherapy could promote the release of neoantigens 153, 154 and reduce immunosuppression in TME, thus improving the effect of immunotherapy 155, 156. Local immunochemotherapy significantly activated neoantigen-specific CD8+ T cells response, which recognized and lysed tumor cells 157. In a mouse tumor model with lousy immunogenicity received radiation, immunogenic gene mutations increased 158. CD4+ and CD8+ T cells could be induced by vaccination with new epitopes encoded by these genes, thus improving the efficacy of radiation therapy, disregarding their ineffectiveness in preventing tumor growth. Additionally, photothermal therapy (PTT) used photothermal agents to produce local hyperthermia, afterward, eliminated tumors. The combination of PTT and cancer vaccines effectively eradicated the sizeable primary tumor, and tumors wholly subsided 159. What's more, the combination therapy exerted a powerful abscopal effect on distant metastases.

### How can the efficacy of neoantigen vaccines be enhanced?

**Figure 4:**
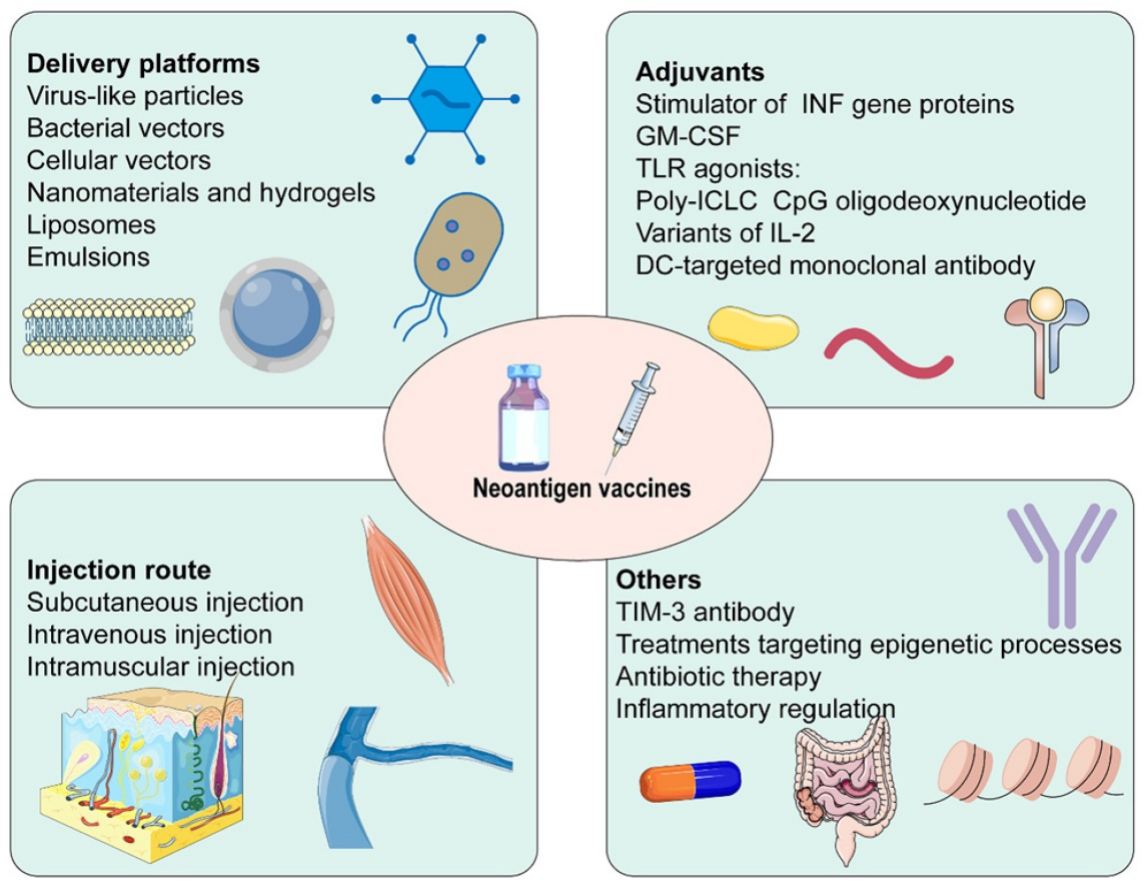
Some factors affect the efficacy of neoantigen vaccines.

The efficacy of cancer vaccines was connected with generating robust T cell responses (Figure 4) 14. Firstly, several neoantigen vaccine platforms have been established in recent years. Virus-like particles increased antibody titers, the efficiency of cross-presentation, as well as the proportion of CD4+ T cells, CD8+ T cells, and effector memory T cells. They could also decrease the proportion of MDSC 160. Similarly, neoantigens expressed by engineered bacterial vectors infiltrated the tumor, resulting in a relative increment in CD4+ and CD8+ T cells and cytokine release 161. Besides, nanomaterials and hydrogels delivered neoantigen vaccines and adjuvants to lymph nodes and APCs in coordination. They promoted the maturation of APCs, and cross-presentation of antigens, and were retained in lymph nodes for a long time, playing a robust and durable anti-tumor effect 162-164. Secondly, adjuvants were critical to successful cancer vaccines. IFN-γ treatment not only increased HLA I and HLA II presenting peptides but also facilitated the expression of IFN-γ induction genes and immune proteasome genes 165. Some adjuvants based on the stimulator of INF genes, such as cGAMP 166, ADU-V16 167, and ADU-V19 168, could trigger INF production, stimulate DCs maturation and antigen cross-presentation. IL-2, the first cytokine approved for clinical cancer treatment, was restricted by the proliferation of Tregs expressing high-affinity IL-2Rα (CD25), which hindered anti-tumor immunity. Several structural variants of IL-2 without binding to IL2Rα have been developed, for example, Bempegaldesleukin (BEMPEG: NKTR-214) 169 and Neoleukin-2 (Neo2/15) 170. A study found that neoantigen vaccination alone was ineffective, BEMPEG monotherapy cured 20% of the mice, and the combination led to tumor regression in half mice. These may be related to the increased level of vaccine-induced T cells and CD8+ T cells infiltration, and the reduced population of Tregs caused by BEMPEG 169. Furthermore, TLR played an essential role in anti-infective immune responses. CPG oligodeoxynucleotides, TLR9 agonists, were efficient vaccine adjuvants, promoting the secretion of IFN-γ, TNF-α, and IL-6, simultaneously activating the expression of T cells 171. Thirdly, the immunosuppressive TME was a significant factor in the efficacy of neoantigen vaccines. TIM-3 antibody noticeably reduced Tregs in tumor tissue and enhanced levels of IFN-γ and IL-12P70. It promoted infiltration of CD8+ T cells and effectively suppressed the progression of HCC in situ with the combination of neoantigen vaccines 172. Fourthly, treatment targeting epigenetic processes upregulated the expression of neoantigen and induced antigen-driven immune response, which provided a promising and appealing strategy for the efficacy enhancement of vaccines 173. Fifthly, the efficacy of the vaccine response was influenced by the injection route. Subcutaneous injection significantly enhanced the ability of neoantigen vaccine delivery to lymph nodes and improved uptake of neoantigen. It stimulated neoantigen-specific T cell responses 7 times and 20 times as much as intramuscular and intravenous injection, respectively 174, 175. Furthermore, the vaccination pathway affected the differentiation of neoantigen-specific CD8+ T cells. Tetramer staining showed that most neoantigen-specific CD8+ T cells were short-lived effector cells after subcutaneous injection, whereas a high proportion was primarily memory precursors effector cells after intravenous injection. Single-cell RNA sequencing showed that intravenous injection induced stem cell-like genes, while subcutaneous injection enriched effect genes. Therefore, intravenous injection had a better anti-tumor response after checkpoint blockade 175. Besides, the development and differentiation of immune cells were linked to the intestinal flora. The decrease in the diversity of the microbiome on account of long-term antibiotic therapy produced a higher tumor-specific immune response, thereafter, performing a greater anti-tumor induced by neoantigen vaccines 176. Finally, inflammatory regulation was a prospective strategy for improving the efficacy of neoantigen immunotherapy 177.

## Challenges

Despite the solid and enduring anti-tumor effect of personalized neoantigen vaccines in some patients, a series of open questions restricted their wide clinical application 134. Firstly, thousands of heterologous gene mutations typically existed in tumor samples. However, only a few fulfilled the eligibility criteria of neoantigen. At present, effective screening methods are still lacking. Secondly, it was widely believed that neoantigen expression led to adaptive immunity and disease suppression. Surprisingly, neoantigen expression contributed to the deterioration of the fibroinflammatory microenvironment that resulted in progression and metastasis in pancreatic ductal adenocarcinoma, which was associated with pathogenic TH17 responses 178. Thirdly, for patients with multifocal tumors, analysis of a single sample might inaccurately capture the complexity of the neoantigen pool. Intrahepatic metastasis (IM) or multicentric occurrence (MO) was a significant feature of HCC. Nonetheless, IM and MO had different genetic susceptibility, clonal structural, and mutation profiles 179. Multi-region sequencing may provide more comprehensive information for neoantigen vaccine design. Fourthly, serious consideration of shared neoantigens was in need. Accumulation of mutations promoted tumorigenesis and ultimately led to tumor invasion and metastasis. Once a malignant tumor developed, a single cancer cell continued to acquire mutations, causing multiple different genomic maps within the tumor. Studies have found that neoantigens in both primary and metastatic tumors were similar in terms of the number and type. Nevertheless, the proportion of shared neoantigens remained modest 180. Optimal therapeutic outcome was not provided by vaccines solely targeting primary tumor neoantigens. Fifthly, in vaccine development, various biometric assay technologies, from genome sequencing to the preparation of personalized neoantigen vaccines, were challenging and costly. These may limit the use of neoantigen vaccines in the population, and some patients may not receive ultimate treatment due to the long vaccine preparation cycle. Further, the frequency of vaccination and the interval of administration of neoantigen vaccines require clinical practice.

## Conclusion

The immune system could attack cancer cells specifically and adapt to the evolving tumor and memory, making immunotherapy the fourth potent weapon in tumor control after surgery, radiotherapy, and chemotherapy. Neoantigens were mutated antigens expressed in tumor tissue specifically but did not exist in normal cells. Sequencing technologies improved the accuracy of neoantigen prediction and identification. Neoantigen vaccines effectively induced the production of tumor-specific T cells without killing normal cells. Syntactic peptides, DNA vaccines, RNA vaccines, and DC vaccines have shown reliable safety, tolerability, and immunogenicity in clinical trials. Whether used alone or in combination with other immunotherapies and conventional therapies, they have shown perfect anti-tumor effects, representing the frontier progress and prospect of cancer treatment. However, due to the loss of neoantigen, the barrier to neoantigen processing and presentation, and the influence of TME, some patients fail to achieve the expected effect of neoantigen vaccine therapy, which is a primary challenge for the application of neoantigen vaccines. Nevertheless, with a better understanding of neoantigens and tumor immunity, we have abundant reasons to believe that the future of neoantigen vaccines is bright in tumor immunotherapy.

## Author contributions

ZQL, XWH, and QD provided direction and guidance throughout the preparation of this manuscript. JXL, ZQL, and QD wrote and edited the manuscript. QD reviewed and made significant revisions to the manuscript. LL, SYW, LBW, XYG, QD, ZKZ, YK, HYL, YLH, and ZQL collected and prepared the related papers. All authors read and approved the final manuscript.

## Figures and Tables

**Table 1:** Advantages and disadvantages of different forms of vaccines

Vaccines type	Advantages	Disadvantages
Peptide	Stable, Safe, Low-complexity antigens, Low toxicity, Easy to transport	Low immunogenicity in the absence of adjuvants, Unrelated immune response due to degradation, Short half-life
DNA	Easy fabrication, Low cost, Polyepitope	Theoretical integration risk, Potential risk of abnormal transcription, Discomfort electroporation
RNA	Less side effects, Low synthesis expenses, Short synthesis cycle, Simultaneous encoding of multiple antigen sequences, Inadequate restriction of MHC haplotypes	Unstable, Innate immunogenicity, Easy degradation by RNases, Inefficient delivery, Harsh storage condition
DC	Low toxicity, Lack of invasive procedures, Potential long-term effects, Conventional antigen presentation pathways	Time-consuming, High cost, Less feasible to produce at large scale

**Table 2:** Clinical trials of personalized neoantigen-based vaccines

ClinicalTrials.gov identifier	Phase	Tumor types	Number of patients	Treatment	Form of vaccine	Status
NCT03359239	I	Urothelial/Bladder Cancer	10	PGV001 with atezolizumab; adjuvant Poly-ICLC	Peptide	Completed
NCT03633110	I/II	Melanoma, NSCLC, SCCHN, RCC or urothelial carcinoma	24	GEN-009 alone or combined with nivolumab or pembrolizumab; adjuvant Poly-ICLC	Peptide	Completed
NCT03645148	I	Pancreatic cancer	7	iNeo-Vac-P01; adjuvant GM-CSF	Peptide	Completed
NCT02454634	I	Grade 3 and 4 IDH1(R132H) + astrocytomas	32	IDH1-vac	Peptide	Completed
NCT01970358	I	Stage IIIB/C or stage IVM1b high-risk melanoma	8	NeoVax; adjuvant Poly-ICLC	Peptide	Completed
NCT02897765	Ib	Advanced melanoma, NSCLC, or bladder cancer	82	NEO-PV-01 plus anti-PD-1; adjuvant Poly-ICLC	Peptide	Completed
NCT03662815	I	Advanced solid tumors	22	iNeo-Vac-P01; adjuvant GM-CSF	Peptide	Active, not recruiting
NCT04799431	I	Pancreatic cancer, colorectal cancer	12	Vaccine and retifanlimab; adjuvant Poly-ICLC	Peptide	Not yet recruiting
NCT05111353	I	Pancreas cancer	30	Vaccine; adjuvant Poly-ICLC	Peptide	Not yet recruiting
NCT04487093	I	NSCLC	20	Vaccine combined with targeted drug	Peptide	Recruiting
NCT03122106	I	Pancreatic cancer	15	Vaccine alone	DNA	Active, not recruiting
NCT03199040	I	TNBC	18	Vaccine alone or combined with durvalumab	DNA	Active, not recruiting
NCT03532217	I	Metastatic hormone-sensitive prostate cancer	19	Vaccine in combination with nivolumab/ipilimumab and PROSTVAC	DNA	Active, not recruiting
NCT03988283	I	Pediatric recurrent brain tumor	10	Vaccine alone	DNA	Not yet recruiting
NCT04015700	I	Glioblastoma	12	Vaccine alone	DNA	Recruiting
NCT04397003	II	Extensive-stage small cell lung cancer	27	Vaccine in combination with durvalumab	DNA	Recruiting
NCT03480152	I/II	Metastatic gastrointestinal cancer	4	mRNA-4650	RNA	Terminated, has results
NCT02035956	I	Stage III-IV melanoma	13	mRNA vaccine alone or combined with PD-1 blockade	RNA	Completed
NCT03289962	Ib	NSCLC, colorectal cancer, melanoma, and breast cancer	29	RO7198457 alone or combined with atezolizumab	RNA	Active, not recruiting
NCT05198752	I	Solid tumor	36	SW1115C3	RNA	Not yet recruiting
NCT00683670	I	Stage III melanoma	3	Vaccine alone	DC	Completed
NCT02956551	I	Advanced lung cancer	12	Vaccine alone or combine with PD-1 inhibitor	DC	Unknown
NCT03171220	I	Solid tumors	6	Vaccine alone	DC	Unknown
NCT03871205	I	Lung cancer	30	Vaccine alone	DC	Unknown
NCT04912765	II	HCC	60	Vaccine and nivolumab	DC	Recruiting

GM-CSF, granulocyte macrophage colony-stimulating factor; NSCLC, non-small cell lung cancer; SCCHN, squamous cell carcinoma of the head and neck; RCC, renal cell carcinoma; TNBC, triple-negative breast cancer; HCC, hepatocellular carcinoma

## Abbreviations

ACT: adoptive T cell therapy
AML: acute myeloid leukemia
APC: antigen presentation cell
CNN: convolutional neural network
DC: dendritic cell
ecDNA: extrachromosomal DNA
EGFR: epidermal growth factor receptor
EGFRvIII: EGFR variant III
HCC: hepatocellular carcinoma
HLA: human leukocyte antigen
ICIs: immune checkpoint inhibitors
IFN-γ: interferon-γ
IM: intrahepatic metastasis
Indels: insertions and deletions
IR: intron retention
K: lysine
MDSC: marrow-derived suppressor cells
M: methionine
MHC: major histocompatibility complex
MM: multiple myeloma
MO: multicentric occurrence
MS: mass spectrometry
MSI-H: microsatellite instability-high
NSCLC: non-small cell lung cancer
nsSNVs: non-synonymous SNVs
PFS: progression-free survival
PTT: photothermal therapy
R132: arginine 132
SNVs: single nucleotide variations
TACE: transcatheter arterial chemoembolization
TAP2: antigen peptide transporter-2
TCR: T cell receptor
Th1: T-helper-1
TILs: tumor-infiltrating lymphocytes
TIM-3: T-cell immunoglobulin and mucin-containing molecule 3
TLR: toll-like receptor
TMB: tumor mutational burden
TME: tumor microenvironment
TNBC: triple-negative breast cancer
TNF: tumor necrosis factor
Tregs: regulatory T cells
WES: whole exon sequencing
